# Numerical and physical modeling of breast cancer based on image fusion and artificial intelligence

**DOI:** 10.1007/s10549-023-07056-1

**Published:** 2023-07-25

**Authors:** Bartosz Dołęga-Kozierowski, Piotr Kasprzak, Michał Lis, Bartłomiej Szynglarewicz, Rafał Matkowski, Marek Sawicki, Mateusz Dymek, Adrianna Szumiejko, Gustavo Carmo, Artur Kwiatkowski, Daniel Grzegorz Soliński, Mariusz Ptak

**Affiliations:** 1 Breast Unit, Department of Breast Imaging, Lower Silesian Oncology, Pulmonology and Hematology Center, Wroclaw, Poland; 2 Burn and Plastic Surgery Department, Ludwik Rydygier Memorial Specialized Hospital in Krakow, Krakow, Poland; 3 Department of Oncology, Faculty of Medicine, Wroclaw Medical University, Wroclaw, Poland; 4 Faculty of Mechanical Engineering, Wroclaw University of Science and Technology, Lukasiewicza 7/9, 50-371 Wroclaw, Poland; 5 Department of Mechanical Engineering, Centre for Mechanical Technology and Automation (TEMA), Campus Universitário de Santiago, University of Aveiro, 3810-193 Aveiro, Portugal; 6 Department of Neurosurgery, Provincial Specialist Hospital in Legnica, Iwaszkiewicza 5, 59-220, Legnica, Poland; 7 Regional Specialist Hospital in Wroclaw, Research and Development Center, Wroclaw, Poland

**Keywords:** Breast cancer, 3D breast modeling, Scanning, Numerical methods, Image fusion, Artificial intelligence

## Abstract

**Purpose:**

The key problem raised in the paper is the change in the position of the breast tumor due to magnetic resonance imaging examinations in the abdominal position relative to the supine position during the surgical procedure. Changing the position of the patient leads to significant deformation of the breast, which leads to the inability to indicate the location of the neoplastic lesion correctly.

**Methods:**

This study outlines a methodological process for treating cancer patients. Pre-qualification assessments are conducted for magnetic resonance imaging (MRI), and 3D scans are taken in three positions: supine with arms raised, supine surgical position (SS), and standing. MRI and standard ultrasonography (USG) imaging are performed, and breast and cancer tissue are segmented from the MRI images. Finite element analysis is used to simulate tissue behavior in different positions, and an artificial neural network is trained to predict tumor dislocation. Based on the model, a 3D-printed breast with a highlighted tumor is manufactured. This computer-aided analysis is used to create a detailed surgical plan, and lumpectomy surgery is performed in the SS. In addition, the geometry of the tumor is presented to the medical staff as a 3D-printed element.

**Results:**

By utilizing a comprehensive range of techniques, including pre-qualification assessment, 3D scanning, MRI and USG imaging, segmentation of breast and cancer tissue, model analysis, image fusion, finite element analysis, artificial neural network training, and additive manufacturing, a detailed surgical plan can be created for performing lumpectomy surgery in the supine surgical position.

**Conclusion:**

The new approach developed for the pre-operative assessment and surgical planning of breast cancer patients has demonstrated significant potential for improving the accuracy and efficacy of surgical procedures. This procedure may also help the pathomorphological justification. Moreover, transparent 3D-printed breast models can benefit breast cancer operation assistance. The physical and computational models can help surgeons visualize the breast and the tumor more accurately and detailedly, allowing them to plan the surgery with greater precision and accuracy.

## Introduction

Breast cancer is the most diagnosed cancer among women worldwide and the fifth leading cause of cancer-related death [1]. The risk factors for developing breast cancer include female sex, age, positive family history of breast or ovarian cancer, genetic mutations, ethnic origin, number of pregnancies and breastfeeding, age at onset of menstruation and at menopause, breast tissue composition, and history of breast cancer or hormone replacement therapy [2].

The GLOBOCAN 2020 database, released by the International Agency for Research on Cancer (IARC), reported 2.3 million new cases of breast cancer [1]. At the same time, age-standardized incidence rates (ASIR) of breast cancer demonstrate a strong correlation with human development index (HDI) scores: clear evidence was provided that the incidence of breast cancer in the most developed countries was significantly higher in younger patients, which makes this an even more pressing issue, both economically and socially [3].

Breast cancer diagnosis and treatment constitute a multistage and interdisciplinary process that involves specialists from various fields, including surgeons, clinical oncologists, radiotherapists, radiologists, pathomorphologists, and psycho-oncologists. The guiding principle of combination therapy is surgical treatment, either currently widely implemented breast-conserving therapy (BCT) or, in specific cases, mastectomy, i.e., complete amputation of the breast. BCT is associated with significantly better esthetic outcomes, reduced psychological distress for the patient and fewer postoperative complications [4]. Although BCT appears much more beneficial to patients, those who have received this treatment often require reoperation with total mastectomy because the primary intervention did not meet the curative surgery criteria [5]. According to the literature, the rate of nonradical BCT resections averages 20% to 30% [6] and reaches as high as 31% to 46% for patients diagnosed with ductal carcinoma in situ (DCIS) [7, 8]. Therefore, the recommended surgical approach for patients with early-stage breast cancer in the guidelines of the European Society for Medical Oncology (ESMO) is based on tumor size, qualification for a specific type of surgery, clinical phenotype and the preferences of the patient. The preparation of an adequate surgical plan for a breast-conserving procedure requires detailed information about the lesion's location and margins, which raises the issue of accurately locating breast lesions on imaging tests.

Because the patient may take different positions during imaging diagnostic tests (standing during MMG or prone during MRI) than her position during surgery (lying on her back with the upper limb in abduction) and because breast tissue movement is influenced by gravity and the position of other body parts, a gold standard in planning the optimal extent of breast resection has not yet been defined.

Ideas for introducing image fusion using the available breast imaging methods (mammography and ultrasound with MRI) to allow for more precise identification of tumor’s location and surgical margins have been appearing in the literature over the past several years. However, lesions detectable only on MRI and missed by other imaging techniques, multifocal breast cancer, and discrepancies in lesion size between particular imaging tests currently remain the greatest challenges. An approach that would enable precise identification of tumor location during diagnostics and surgery has not yet been created, due to a number of difficulties:

- Deformation of breast tissue in the MRI breast coil and difficulties reproducing this deformation pattern in the surgical position
- Variability of breast tissue composition (ratio of fatty tissue to glandular/fibrous tissue)
- Variability of water retention in a given patient’s breast depending on the stage of her menstrual cycle

A model of breast image fusion would lead to countless benefits for all specialists involved in the diagnostics and treatment of breast cancer, as well as numerous advantages for the patients. A breast image fusion model would have numerous benefits for both clinicians and patients in the diagnosis and treatment of breast cancer. It would allow for effective staging, pre-operative planning, and the identification of additional lesions. Surgeons could choose an optimal breast reconstruction technique and plan ahead for the procedure, resulting in a better quality of life after treatment. This planning approach could also be used for targeted examinations, a training model for further education, and improved communication with patients. Radiologists could use 3D printing to present realistic and accessible radiological data in the form of a real, accurate 3D model of a particular pathology [2]. The economic aspects of improved communication, surgical planning, and choosing BCT whenever possible could lead to shorter preparation and procedure times, reduced drug use, a faster learning curve for new surgeons, and fewer postoperative complications and treatment costs. Overall, a breast image fusion model could greatly improve the diagnosis and treatment of breast cancer for all those involved.

## State of the art

Many studies [9–11] only conduct analyses of breast movements while performing activities in an upright position, i.e., walking on a treadmill, running, or ascending and descending on an elevation. The female breast is essentially composed of four structures: lobules or glands, milk ducts, fat and connective tissue and each has different mechanical properties [12]. For this reason, when creating FE (Finite Element) models, certain simplifications are adopted.

Chen et al. [9] created a breast model that consisted of a thorax, two breasts and three layers of skin with different mechanical properties (density, Young’s modulus, Poisson’s ratio, and shear modulus). An established nonlinear finite element method (FEM) has been utilized to simulate the motion of human breasts based on 3D photogrammetric surface models and biomechanical properties that were tested through finite element method and motion analysis.

Naser et al. [13] proposed a simple geometry of the woman’s breast. The idea of the study is to show the finite element (FE) capabilities in predicting the shape under specific loads. Characterizing the biomechanical performance of breast mass types using force-strain difference curves presented a high variance singularity between the breast mass types. Based on previous studies [14–17], authors assumed all tissues to be incompressible, homogenous, isotropic, and have nonlinear elastic properties. Vavourakis et al. [18] presents in his paper a three-dimensional surgical simulator for computer-aided surgical planning breast-conserving therapy (BCT) of early-stage breast cancer patients. Authors attempt to model—in an integrative manner—breast tissue biomechanics and physiological soft-tissue recovery with MRI and 3dMD surface acquisition. Dufaye et al. [19] present a virtual deformable breast model of a representative volunteer whose geometry is constructed from MR data. The study confirms that the mechanical properties of the breast skin play an important role in explaining the changes associated with radiotherapy, tissue expansion, and breast reconstruction surgery. The proposed by the authors numerical modeling takes into account the main constituents such as skin, fat, glands or fibers and suspensory ligaments of Cooper, responsible for the deformability of breast tissues under the effect of gravity.

In summary, the discussed studies present an overview of the numerical modeling used in estimating breast strain in different loading conditions (prone, supine, and standing positions). We observed that the majority of articles implement the inverse approach. In our opinion, this is an excellent way to obtain the initial breast geometry. The inverse approach is a method to obtain a stress-free (i.e., state without gravity) tissue geometry. Thus, it is possible to apply new boundary conditions and verify the strains acting on the breast. In other words, it is impossible to simulate the geometry change from prone (i.e., MRI position) to supine (i.e., surgery position) without first simulating the stress-free position.

In the studies, it is observed that there is a huge missing part, which is the cancer tissue position and geometry change. Mammography or MRI usually characterizes the cancer diagnosis. Both methods have a different position than the one during surgery. The woman’s breast is characterized by various tissues, mainly fat, glandular tissue, ligaments, blood vessels and nerves. Finally, while adapting the optimum surgical plan, the surgeon has to decide where the tumor is oriented after the position change. This brings us to the different simulations scope. The idea would be not to focus only on the outer geometry but on the cancerous tissue displacement. Thanks to that, the BCTs will be more efficient, and the reoperation percentage will decrease. Presumably, different numerical methods, such as smoothed particle hydrodynamics (SPH), will come in handy. The SPH is dedicated mainly to fluid and soil representation, but it might be an excellent fit for the fat tissue [20].

Artificial neural networks are considered a promising computational technology to resolve intricate issues that cannot be tackled with conventional approaches, due to their ability to learn from experience and generalize [21]. Nevertheless, when confronted with a complicated dataset, various neural classifiers usually generate distinct generalizations by setting different boundaries. The diversity of outcomes is significantly influenced by several factors such as the neural network's architecture, learning mode (supervised or unsupervised), network structure (number of layers and hidden nodes, type of activation functions, and connectivity degree), training parameters (weights initialization, learning rates, training epochs), and other relevant aspects. In their study, Wu et al. [21] examined the challenge of classifying breast lesions as benign or malignant, considering the binary classification. The aim of the study was to enhance the ratio of successful biopsies by developing computer-aided diagnosis techniques and systems, which can provide valuable assistance to radiologists and physicians in the process of screening and diagnosis. In a different study [22], the use of artificial neural networks has been implemented to categorize mammographic masses with the purpose of detecting and diagnosing breast cancer in its early stages. The authors of this paper employ various fusion methods, both fixed and trained, to detect potentially concerning lesions.

## Methodology

**Fig. 1:**
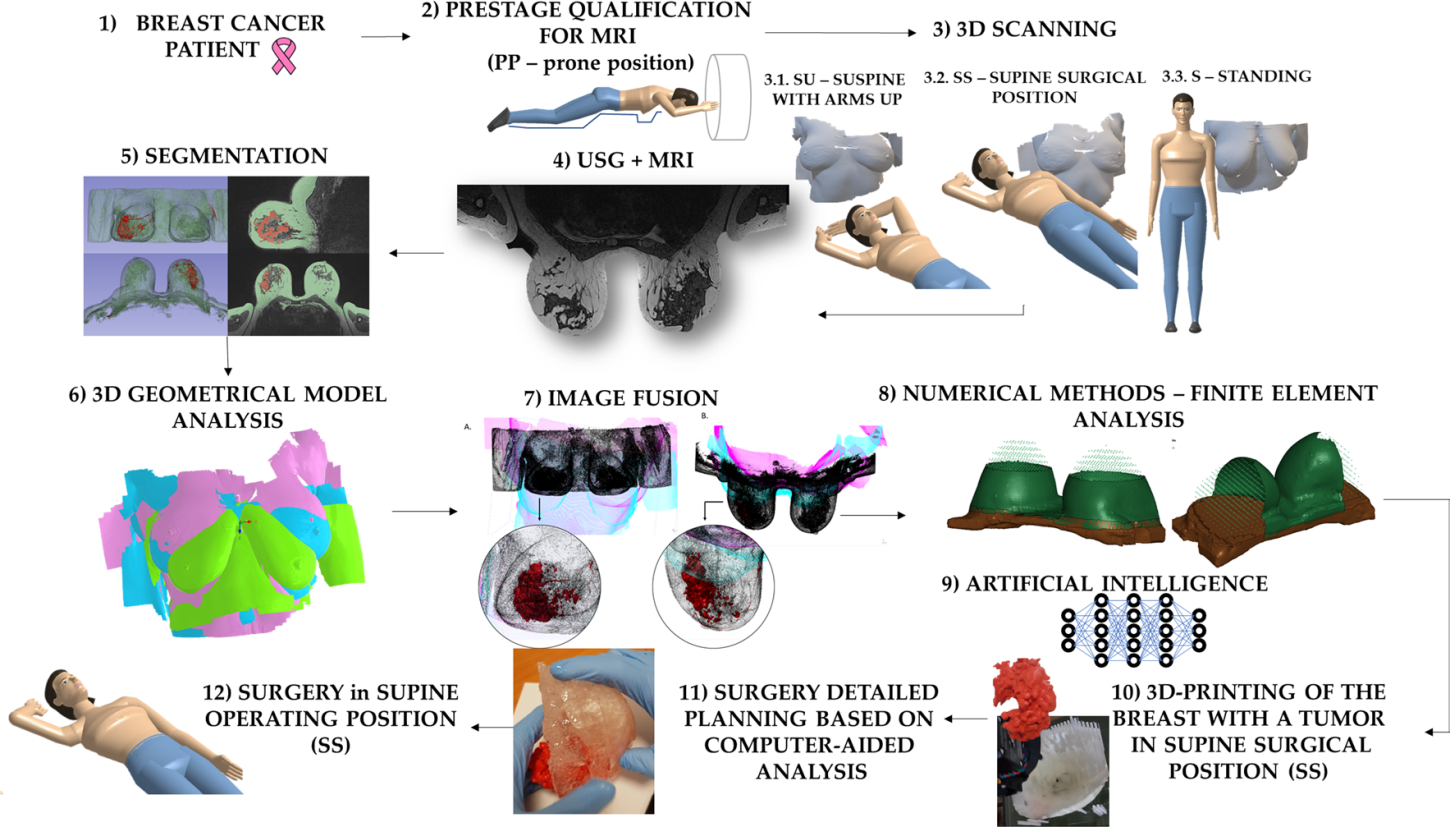
Methodological process for the breast cancer patients treatment

A new approach was developed for the pre-operative assessment and surgical planning of lobular breast cancer patients. The process involves pre-qualification assessment, 3D scanning in three different positions, MRI and USG imaging, breast and cancer tissue segmentation, model analysis, image fusion, finite element analysis, artificial neural network training, STL (stereolithography) model generation, and finally, 3D printing (detailed parameters–see Appendix). Based on the computer-aided analysis, a detailed surgical plan is created for performing lumpectomy surgery in the supine surgical position. The detailed methodology, depicted in Fig. 1, is as follows:

1. Admission of a breast cancer patient to an oncology clinic.
2. Conducting a pre-qualification assessment of patients for magnetic resonance imaging.
3. Taking 3D scans of patients in three different positions: 3.1. supine position with both arms raised up (SU), 3.2. supine position with the arm raised from the breast side with the tumor (forming a 90-degree angle between the arm and the torso—supine surgical position (SS), 3.3. standing with arms resting freely along the body (S).
4. Conducting MRI in the prone position and standard ultrasonography (USG) imaging.
5. Segmenting breast tissue and cancer tissue from MRI imaging.
6. Making model analysis from 3D scanning.
7. Performing image fusion of segmented MRI and 3D models.
8. Conducting finite element analysis using numerical methods to simulate the behavior of breast tissue and the tumor in different positions.
9. Training an artificial neural network to predict tumor dislocation in different positions using artificial intelligence.
10. Additive manufacturing: 3D printed breast model in supine surgical position (SS) with the highlighted tumor – STL file format generated based on iterations #8 and #9.
11. Detailed surgical plan based on computer-aided analysis and 3D physical model.
12. Performing lumpectomy surgery in a supine surgical position (SS).

MRI of the breast was performed on the Magnetom Avanto Tim Dot 1.5 T (Siemens Healthcare, Erlangen, Germany) with a compatible 18-channel diagnostic breast coil in the prone position and in a supine position using a flexible body surface 16-channel coil. Imaging was performed within 14 days of the core needle biopsy. For all axial plane acquisitions in prone position, the phase encoding direction was performed from right to left to limit artifacts repeating cardiac and respiratory movement. Additionally, movement artifacts were eliminated by the “Motion Correction” function. The tests were conducted according to protocol described in Appendix 1 with 30 min gap between two scannings.

Moreover, vitamin D tracers can be fixed onto the skin at the level of the suprasternal notch and approximately 10 cm below in order to establish additional reference points to be used in further imaging. Breast MRI exams were analyzed by independent radiology specialists (double reading) using the Siemens software tool (Brevis MRI—Siemens Helthineers Erlangen Germany), and all lesions were evaluated by the American College of Radiology—BIRADS breast MRI lexicon (Fifth Edition).

### Segmentation

The segmentation of images is a procedure to select certain areas of an image, usually corresponding to a certain anatomical structure or lesion. This process is commonly implemented by the medical community for a better view of a certain structure. For this work, the segmentation is performed on two separate sets of medical images obtained by MRI. The first set of medical images displays the contour and overall volumetry of both breasts. The second set of medical images involves a contrast that reveals cancer tissue. The segmentation of both sets of images allows an overlapping 3D structure that reveals the exact location of the tumors in relation to the position in which the MRI was obtained. This is possible since both MRI sets were obtained without the patient moving, meaning that the reference system from one set to the other was identical.

**Fig. 2:**
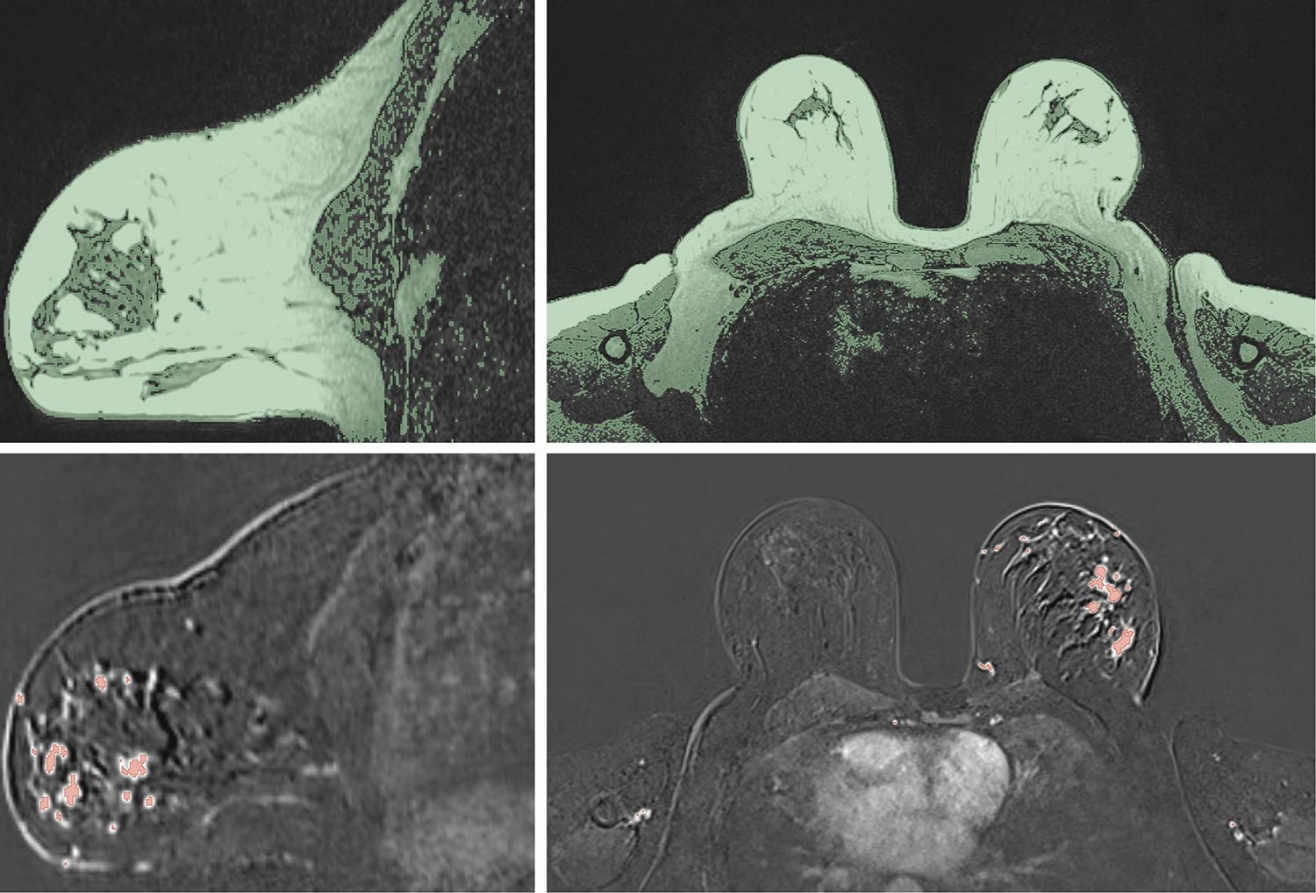
Example of the segmentation results for the breast tissue (top) and cancer tissue (bottom)

The open-source software 3D Slicer (Slicer, 2023) was used to perform all segmentations. After uploading the appropriate sets of medical images, the segmentation is rather straightforward. The MRI scan containing the breast volumetry displays a greyscale contour, in which the user obtains the final geometry by performing a threshold segmentation (Fig. 2). This process selects pixel groups that meet a certain threshold of greyscale value and translating those pixels into 3D representative units named voxels. The representation of the voxels allows the user to observe the final segmented structure.

**Fig. 3:**
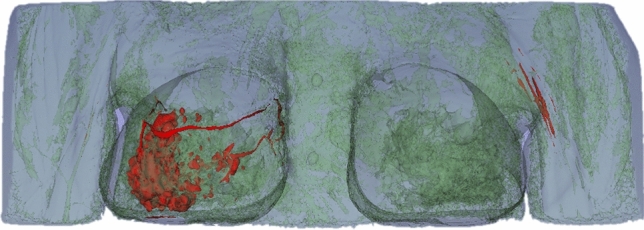
Example of a 3D segmentation result model, displaying the dispersion of cancer tissue

The set of images containing the contrast on cancer tissue allows the user to select pixel groups close to the color white, since the contrast differentiates it from the rest of the breast tissue (Fig. 2). After processing the first two sets of medical images, the threshold values were recorded and used for the rest of the sets. Overall, the values did not differ significantly between set groups. Figure 3 illustrates the final 3D structure obtained for patient 2, where it is visible the cancer tissue dispersion in the patient’s body.

### Image fusion method

The structured-light 3D scanner was used in order to obtain surface models of the patients’ breasts [23]. A professional handheld scanner designed to digitize large objects in a relatively short time was used to provide the comfort for the patients. Thanks to the external camera mounted on the scanner, we were able to scan the breast and chest geometry along with textures in full color. The handheld mode used with the camera enabled obtaining 30 fps, ~ 1.5 million points per second with an accuracy of 0.1–0.4 mm (depending on the scanning distance), which was sufficient for this approach. The scanner projected the visible line pattern or QR code-like pattern depending of the method used. However, in every mode the scanner was projecting the high intensity lighting with some stroboscopic effect—thus the patients were instructed to close the eyes during the scanning process if they feel unconformable.

**Fig. 4:**
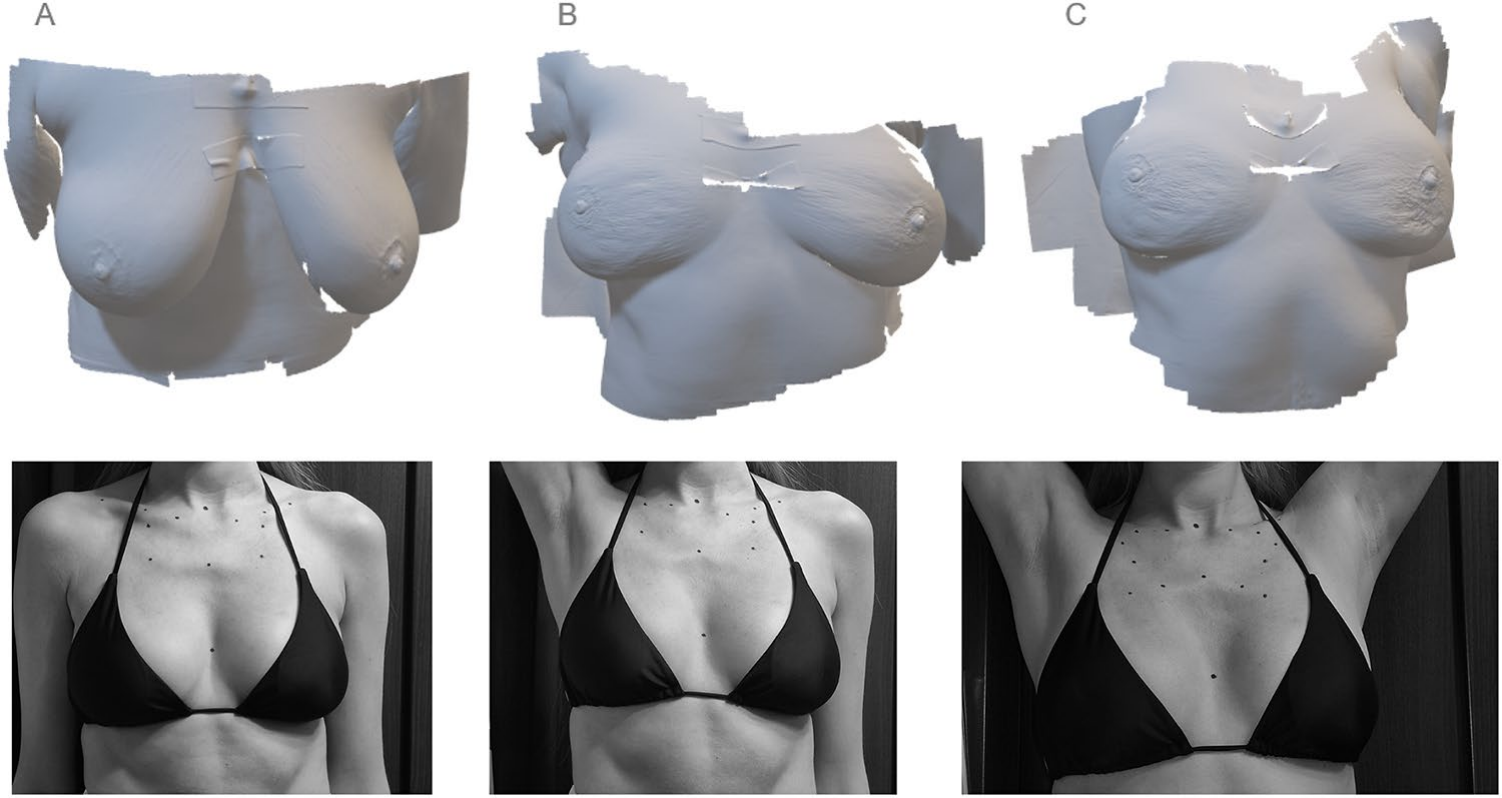
Example of a 3D scans (upper row) of a patient in different positions (lower row): **A** standing with arms resting freely along the body (S), **B** supine surgical position (SS) with the arm raised from the breast side with the tumor change, **C** supine position

Each of the 10 patients was scanned in three different positions (Fig. 4)—standing with arms resting freely along the body (S), supine surgical position (SS) with the arm raised from the breast side with the tumor change, and supine position with both arms up (SU).

Two vitamin D tablets were attached to the patient's body at the suprasternal notch and approximately 10 cm below to establish additional reference points. The average scanning time in one position was approximately 3 min with additional 4–7 min for data processing. Thus, the 3-position setup 3D scanning for each woman took around 25 min in total.

**Fig. 5:**
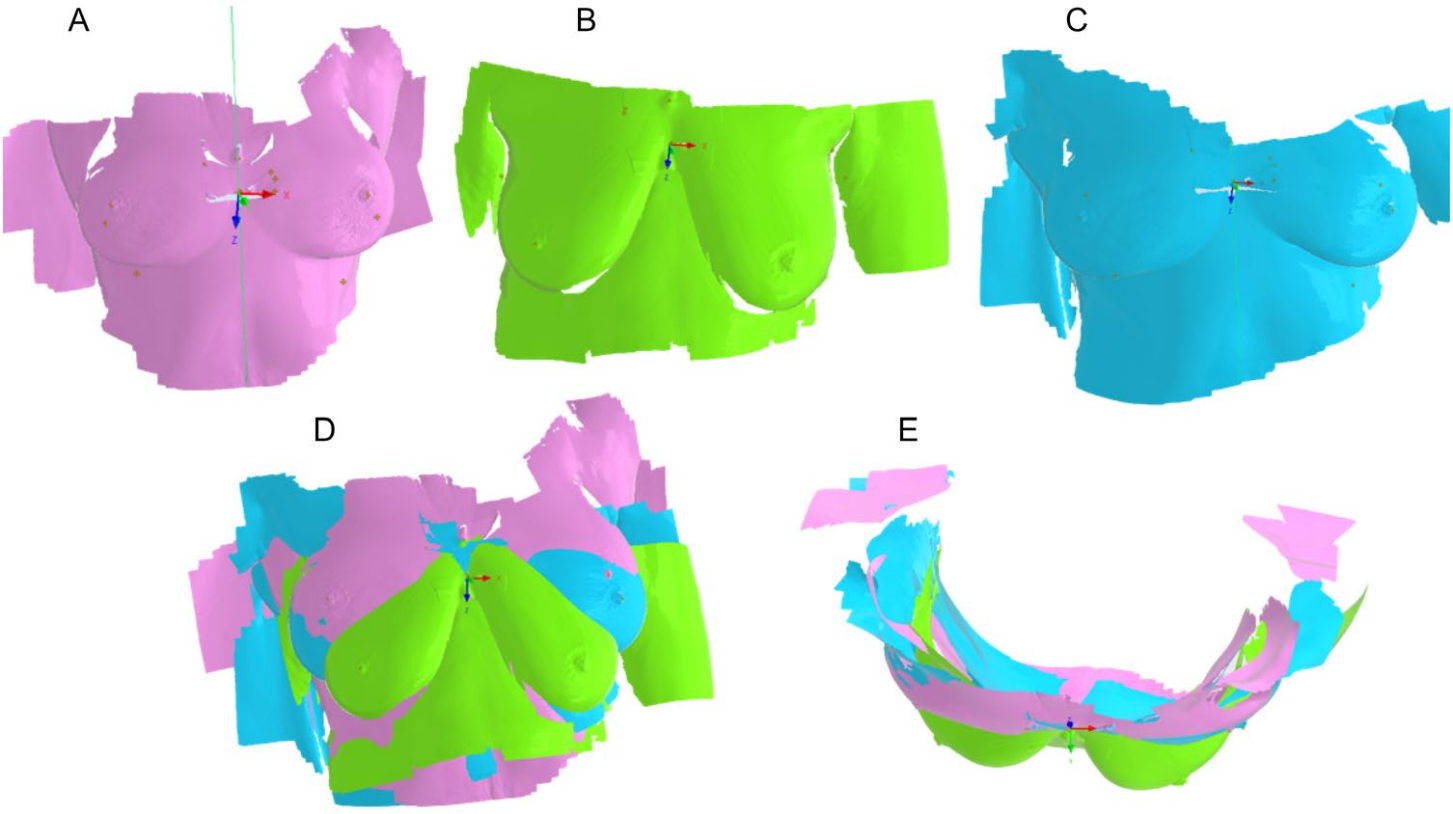
Example of the process of aligning the 3D scans. Images (1**A**–1**C**) show the position of the local coordinate systems in the vicinity of the lower tablet and how the axes pass through the two reference points. (1**A**)—position SU; (1**B**)—position S; (1**C**)—position SS. Images (2**A**, **B**) show all three positions in relation to each other; (2**A**)—frontal plane view; (2**B**)—transverse plane view

**Fig. 6:**
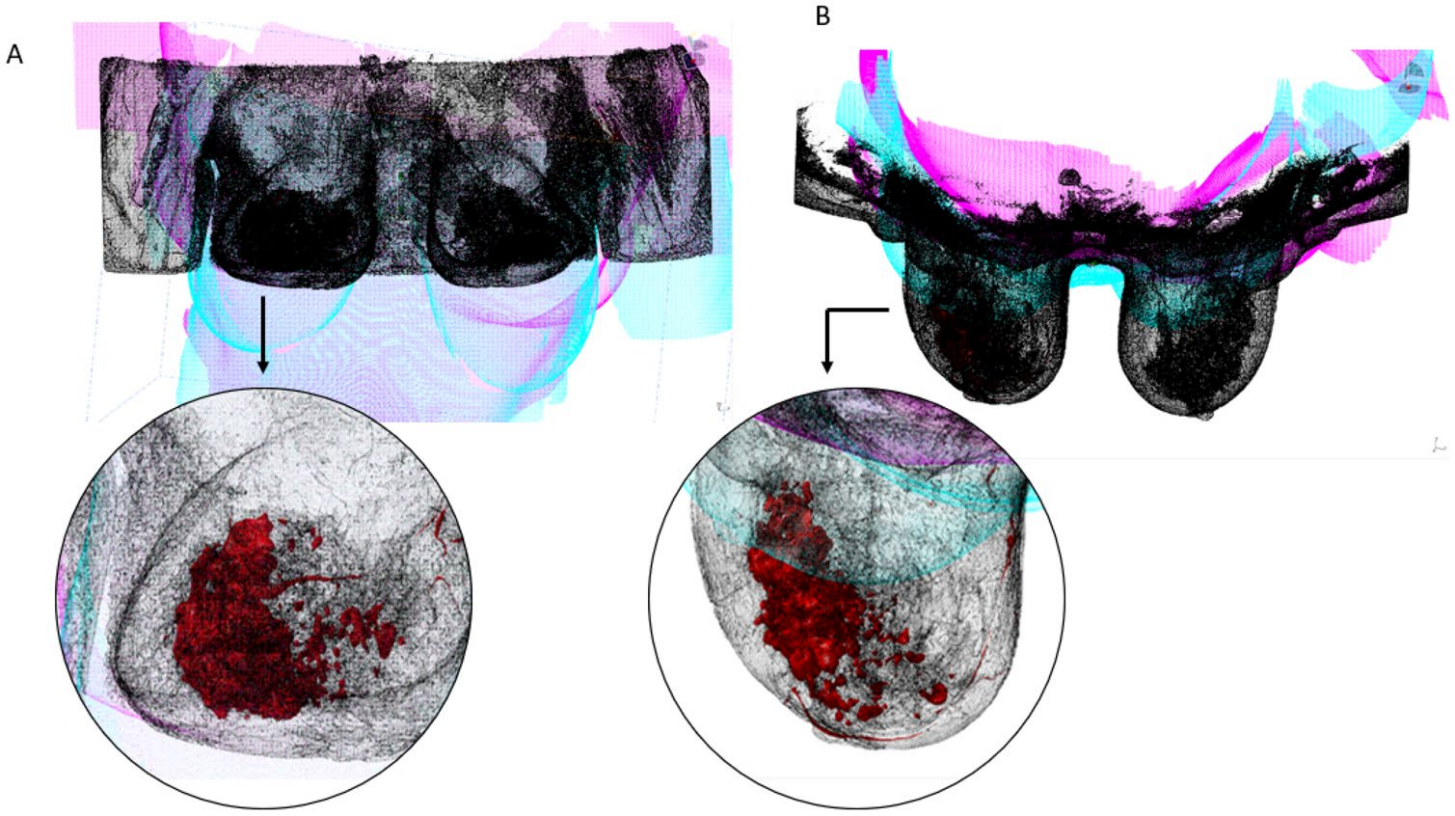
Example of a process of aligning breast models after segmentation (black model—whole breast, red—segmented cancer tissue) with 3D scans aligned in a local coordinate system. **A**—frontal plane view; **B**—transverse plane view

All 3 surface models were processed using CATIA v5. On each model the middle distance between the tablets were marked with a point, and then connected by a line. Operating position (SS) cloud of points was fixed and used as a reference going forward. A local coordinate system was placed in geometrical midpoint of the lower vitamin D tablet due to better visibility and scanning precision. z-axis was fixed in accordance with the direction of gravity and the x- and y-axes were oriented in frontal and sagittal planes, respectively. The S and SU models were linked at the location of the bottom tablet (and thus the location of the local coordinate system), and the lines going through the reference points overlapped. In the next step the models were adjusted by rotation around the z-axis of the local coordinate system (Fig. 5). The process of aligning breast models after segmentation is depicted in Fig. 6.

### Machine learning

**Fig. 7:**
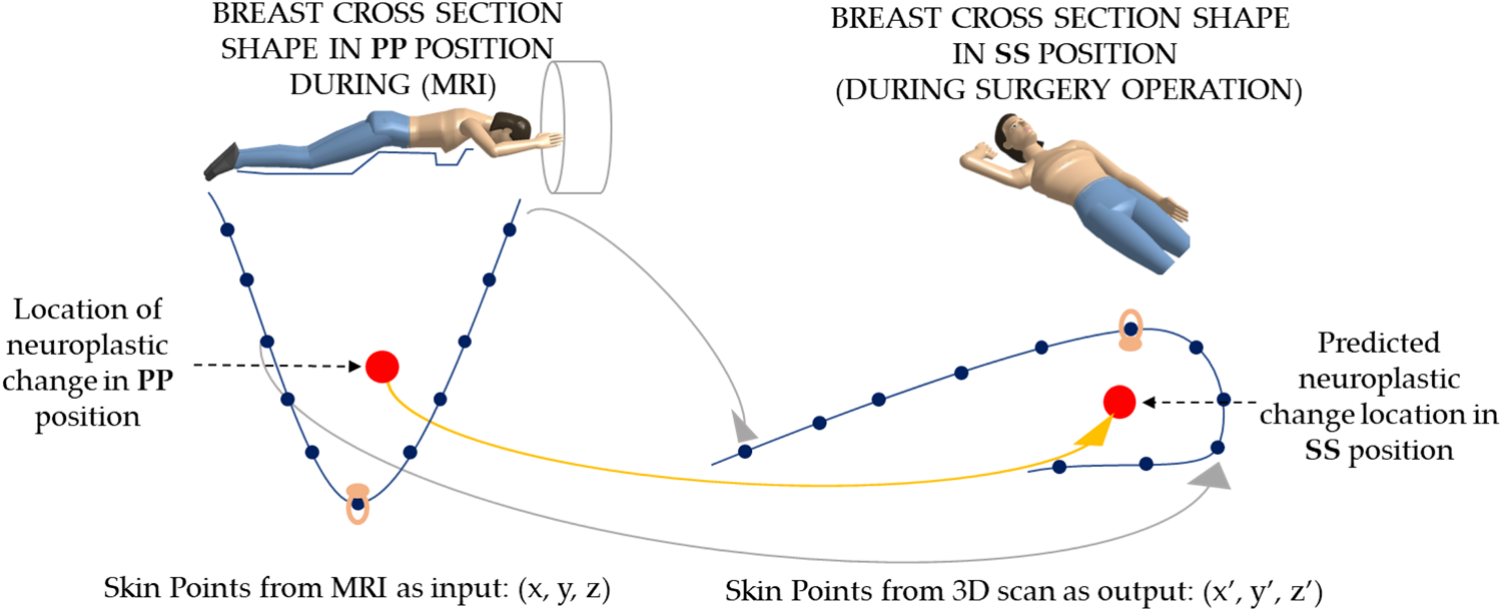
Conceptual of deformation calculation with deep neural network model

AI-based techniques have the potential to revolutionize the prediction of large deformations, especially for human tissue materials, where highly nonlinear material behavior is common (Fig. 7). Accurate prediction of tissue deformation behavior can significantly improve the accuracy of surgical planning, simulations, and personalized medicine. However, more research is needed to address the challenges associated with developing and validating AI-based models for predicting the deformation of human tissue materials.

In this case, numerical models based on FEM might be ineffective in terms of the time-consuming process of obtaining geometry of patient’s breast, preparing numerical model, performing simulation and retrieving results for medical staff. This time comfort cannot always be assured and overloading of cases in short time might lead to bottle-neck problem. One solution might be introducing an AI model based on a deep neural network for obtaining a deformation model of a patient’s breast. Considering the scenario, based on assumptions of personalized medicine, that every single case is treated individually, it required possessing medical data and retrieving it into numerical form. Lots of information from medical imaging techniques can overwhelm AI algorithms—thus reducing the set of information is needed. On the other hand, the description of the problem assumes that the patient’s MRI is performed in the abdominal position while surgery is performed in the supine position (Fig. 7.). Therefore, the location of the tumor inside the breast can be hard to find during surgery operation, moreover for the complex shape of cancer-affected tissue. Thus the shape of the breast is significantly deformed. AI models have an advantage over FE models. For a patient who is examined in MRI in a prone position with vit-D caps for reference points and then placed in a supine position like for surgery and scanned with 3D scanner first, points on the breast skin might be used as trackers between the positions (compare with Fig. 1). This allows training AI models with real input points and real output points. While FEA models can only use real input points and no tuning procedure between the result and real output points is made.

At this stage, the assumption is that only points on the skin could be precisely extracted from MRI examination and 3D scanning. Deep Neural Network (DNN) model has been used in preliminary studies with loss function defined as Root Mean Square Error. The model trained with such inputs obtained over 80% of accuracy for points on the skin. However, at this stage, it should be noted that the position of neoplastic change in the breast is hard to verify. Authors proposed additional MRI in the Supine Surgical position for several patients, un-fortunately neoplastic changes are almost impossible to recognize in this position. Therefore, only post-surgery validation of neoplastic change could be done. In the future, pre-operative ultrasonography might be used to validate the tumor position and an even higher accuracy of the DNN model will be obtained. At this stage, on particular aspect should be raised. These assumptions seem to be valid for all kinds of neoplastic changes, which have tissue mechanical properties similar to breast tissue. However, for much more stiff or attached-to-skin neoplastic changes, this approach has to be tuned and carefully validated.

**Fig. 8:**
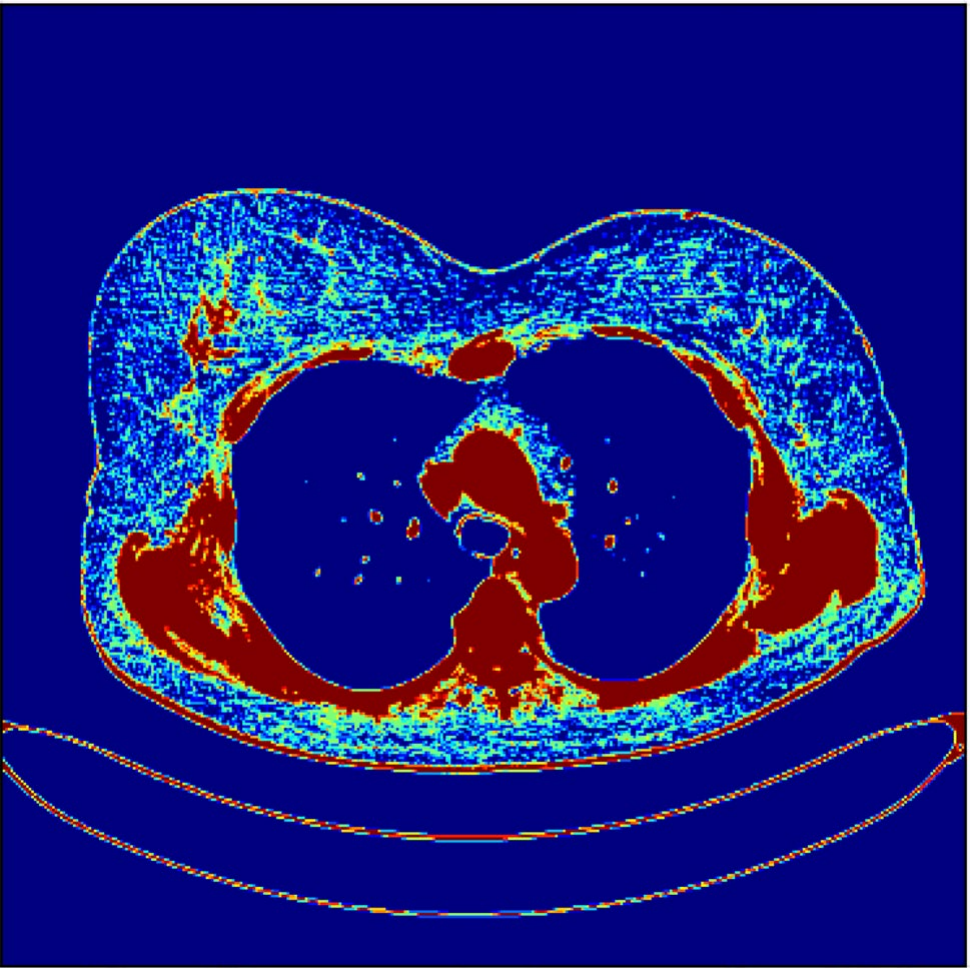
Machine Learning approach for neoplastic change extraction—in red color: the area with a statistically higher level of tissue density than average breast tissue

Moreover, AI and ML (Machine Learning) models can be used in the automatic recognition of neoplastic changes in breast tissue. Figure 8 shows the approach to direct clustering of raw MRI files with SVM model for neuroplastic changes. Further works show that using the predefined mask of Region of Interest (ROI) significantly reduces False Positive regions such as the chest of the spine. In other words, the figure below depicts the area with a statistically higher level of tissue density than average breast tissue. The background blue color indicates zero level, while the red describes a 50% or more statistical chance that this region has higher tissue density. Thus the spine, rib cage, and other structures are shown. It should be noted that this approach is obtained in an automated way. In the future, an automatically generated mask of ROI is assumed to incorporate this kind of extraction.

## Conclusion

Breast cancer remains a significant global health challenge with many risk factors, and early detection is critical. The multistage and interdisciplinary process of breast cancer diagnosis and treatment requires accurate and precise identification of the tumor’s location and margins. The use of image fusion, which combines available imaging techniques, can provide a three-dimensional model that allows effective staging and pre-operative planning, resulting in better surgical outcomes, reduced complications, and improved quality of life for patients. The benefits of a 3D model extend beyond surgery, including improved communication with patients, training opportunities for oncoplastic procedures, and better preparation of histopathological specimens. With the continued development and implementation of image fusion technology, patients with breast cancer can receive optimal care and outcomes. However, there are still limitations in current studies, such as neglecting the mechanical properties of the skin and muscle. Further research is needed to fully utilize the potential of numerical methods in this field. Nonetheless, the studies discussed a valuable contribution toward improving surgical planning and predicting the outcome of breast-conserving therapy.

AI-based techniques can potentially revolutionize the prediction of large deformations in human tissue materials, which is particularly relevant for surgical planning, simulations, and personalized medicine. However, challenges associated with the development and validation of AI-based models require further research. In particular, using numerical models based on FEA may not always be practical for medical personnel due to the time-consuming process of obtaining the geometry of the patient’s breast, preparing the numerical model, performing the simulation, and retrieving the results. An AI model based on deep neural networks may offer a solution to this problem. Additionally, reducing medical data and using real input and output points for training AI models can improve the accuracy of predictions. The surgeon can use the 3D-printed breast model to plan the surgery, including the location and size of the incision and the removal of the tumor. The model can also educate patients about their condition and the surgical procedure.

## Figures and Tables

**Fig. 9:**
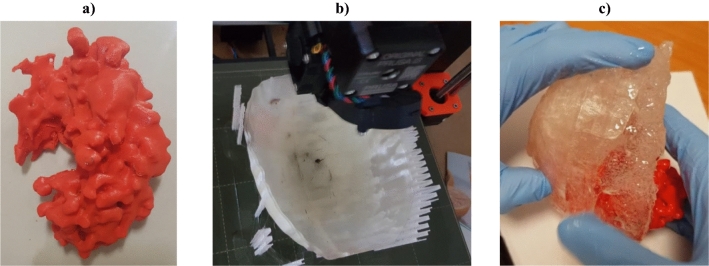
Additive manufacturing (3D-pritining) outputs: **a** cancer lesions **b** outer breast mould **c** resin model with positioned cancer lesion

## Appendix 1

The protocol for MRI examination:

1. Prone position.

T1 HR —slice thickness, 0.7 mm [voxel size: 0.7 × 0.7 × 0.7 mm; SNR (signal–noise ratio), 1.00]; slices per slab, 208; TR, 5.64 ms/TE, 5.64 ms; FoV read, 250 mm; FoV phase, 169.3 and a total acquisition time = 2 min 28 s.

T2—slicethickness, 2 mm (voxel size: 0.5 × 0.5 × 2.0 mm; SNR, 1.00); slices, 57; TR, 6,670 ms/TE, 100 ms; FoV read, 250 mm; FoV phase, and a total acquisition time = 2 min 53 s.

TIRM (Turbo Invertion Recovery Magnitude)—slice thickness, 2 mm (voxel size: 0.7 × 0.7 × 2.0 mm; SNR, 1.00); slices, 57; TR, 7,850 ms/TE, 63 ms; FoV read, 250 mm; FoV phase and a total acquisition time = 3 min 24 s.

T1 3D dynamic—matrix, 389 × 256; slice thickness, 1 mm (voxel size: 1 × 1 × 1 mm; SNR, 1.00); slices per slab, 144; TR, 4.42 ms/TE, 1.7 ms; flip angle, 10°; acquisition time of each phase, approximately 55 s (one phase before contrast, six phases after contrast injection). The dynamic test was performed with the administration of the contrast agent Dotarem (gadoterate meglumine) at the dose of 0.1 mmol/kg and the flow of 2 ml/s, followed by a rinse with 30 ml of NaCl.

DWI—b-value, 50/400/800 s/mm2; slice thickness, 3 mm (voxel size: 1.3 × 1.3 × 3.0 mm; SNR, 1.00); slice numbers, n = 45; TR, 6300 ms/TE, 70 ms; slice gap, 0.6 mm; and a total acquisition time = 3min22s.

(2) Supine position.

T1 HR—slice thickness, 0.7 mm [voxel size: 0.7 × 0.7 × 0.7 mm; SNR (signal–noise ratio), 1.00]; slices per slab, 240; TR, 5.64 ms/TE, 2,74 ms; FoV read, 250 mm; FoV phase, 169.3 and a total acquisition time = 4 min 07 s.

T1 3D dynamic —slice thickness, 1 mm (voxel size: 1,2 × 1,2 × 1 mm; SNR, 1.00); slices per slab, 160; TR, 4.49 ms/TE, 2,16 ms; FoV read, 380 mm; FoV phase, 81,3 acquisition time of each phase, approximately 55 s (one phase before contrast, two phases after contrast injection). The dynamic test was performed with the administration of the contrast agent Dotarem (gadoterate meglumine) at the dose of 0.1 mmol/kg and the flow of 2 ml/s, followed by a rinse with 30 ml of NaCl.

## Appendix 2

A 3D-printed model of the lobular breast cancer with non-mass enhancement morphology and regional distribution was prepared based on contrast-enhanced MR imaging. Using 3D Slicer software cancer, pectoralis major fascia, and skin of the mammary gland were included in the model, excluding blood vessels and other contrast-enhancing tissues. The models were exported in *.stl format to the Shapr3D program (Shapr3D Zrt. Budapest, Hungary) and 3D printing dedicated slicer software, where supports were added and 3D visualizations were made. Cancer lesions were printed with a red polylactide filament, and the circumference of the breast was printed with a transparent polycarbonate-based filament in fused filament fabrication technology using 3D printers (Ultimaker 2 + ; Ultimaker B.V. Zaltbommel, Netherlands; and Original Prusa i3 MK3S + , Prusa Research a.s., Prague, Czech Republic). Parts of the models were accurately positioned and poured with a transparent, flexible polyurethane resin. The outputs are depicted in Fig. 9 below.

## Abbreviations

AI: Artificial intelligence
BCT: Breast-conserving therapy
CAD: Computer-aided design
CAE: Computer-aided engineering
DNN: Deep neural network
FE: Finite element
FEA: Finite element analysis
FEM: Finite element method
MRI: Magnetic resonance imaging
ROI: Region of interest
S: Standing position
SS: Supine surgical position
SU: Supine position with arms raised (up)
SVM: Support vector machine
USG: Ultrasonography
